# Supplementary material for: Graphite-protected CsPbBr3 perovskite photoanodes functionalised with water oxidation catalyst for oxygen evolution in water
Source: Nat Commun. 2019 May 8;10:2097. doi: 10.1038/s41467-019-10124-0 (PMC6506520; doi:10.1038/s41467-019-10124-0)
Supplement: Supplementary file 1 — Supplementary Information [file 41467_2019_10124_MOESM1_ESM.pdf]

Graphite-protected $CsPbBr_3$ Perovskite Photoanodes Functionalised with Water Oxidation Catalyst for Oxygen Evolution in Water

Poli et al.

Supplementary Information

Supplementary Information contains:

- Supplementary Figures 1-28
- Supplementary Tables 1-2
- Supplementary Notes 1-5
- Supplementary Discussion
- Supplementary References

Supplementary Figures

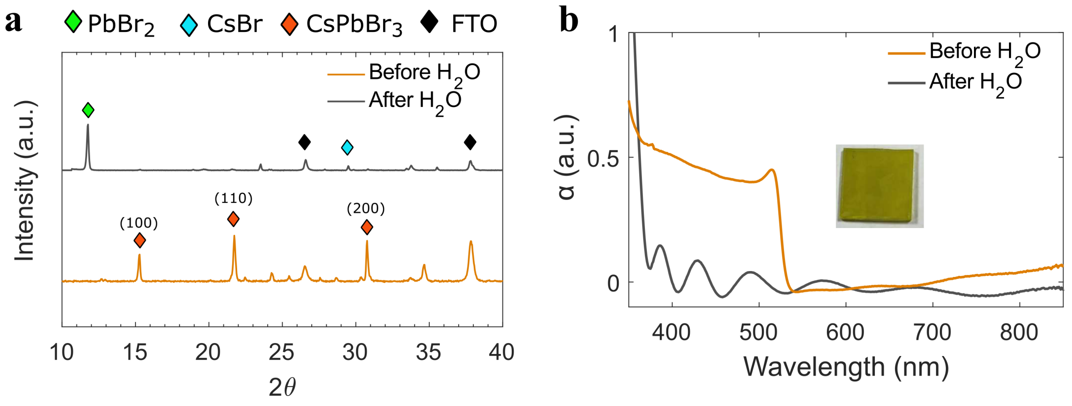

Supplementary Figure 1: Instability of $CsPbBr_3$ in water. a XRD patterns of $CsPbBr_3$ films before and after immersion in water for 1 s. **b** UV-Vis spectra of $CsPbBr_3$ films before and after immersion in water for 1 s; Picture showing the as prepared $CsPbBr_3$ film.

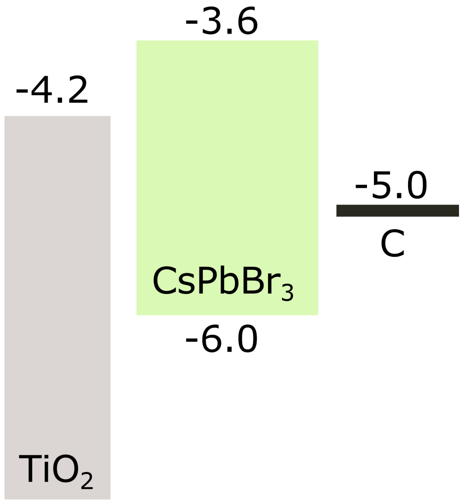

Supplementary Figure 2: Energy band diagram of $CsPbBr_3$-based devices. Schematic of the energy band diagram of $TiO_2|CsPbBr_3|$m-carbon stack used as photoanode in a three-electrode PEC system.

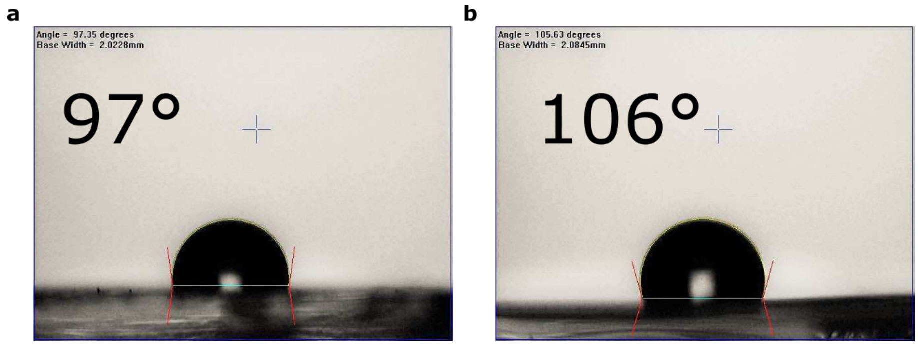

Supplementary Figure 3: Hydrophobicity of the mesoporous carbon HTL. Water contact angle of mesoporous carbon layer **a** doctor bladed and **b** screen printed onto FTO-coated glass.

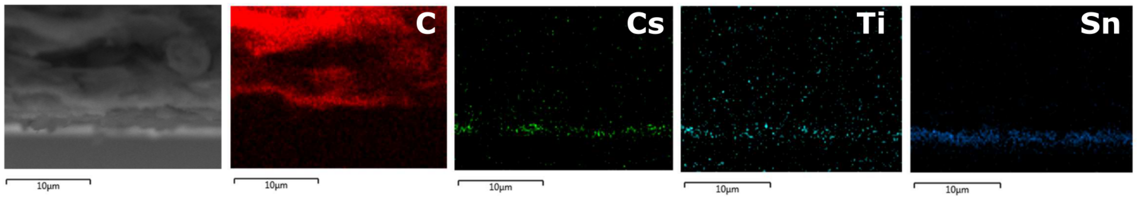

Supplementary Figure 4: Elemental composition of TiO_2|$CsPbBr_3$|m-c. Energy dispersive X-ray (EDX) mapping of the as-prepared TiO_2|$CsPbBr_3$|m-c structure. Red colour indicates C (m-C top contact), green colour is Cs ($CsPbBr_3$ absorber layer), light blue colour is Ti (TiO_2 ETM) and blue colour is Sn (FTO coated glass).

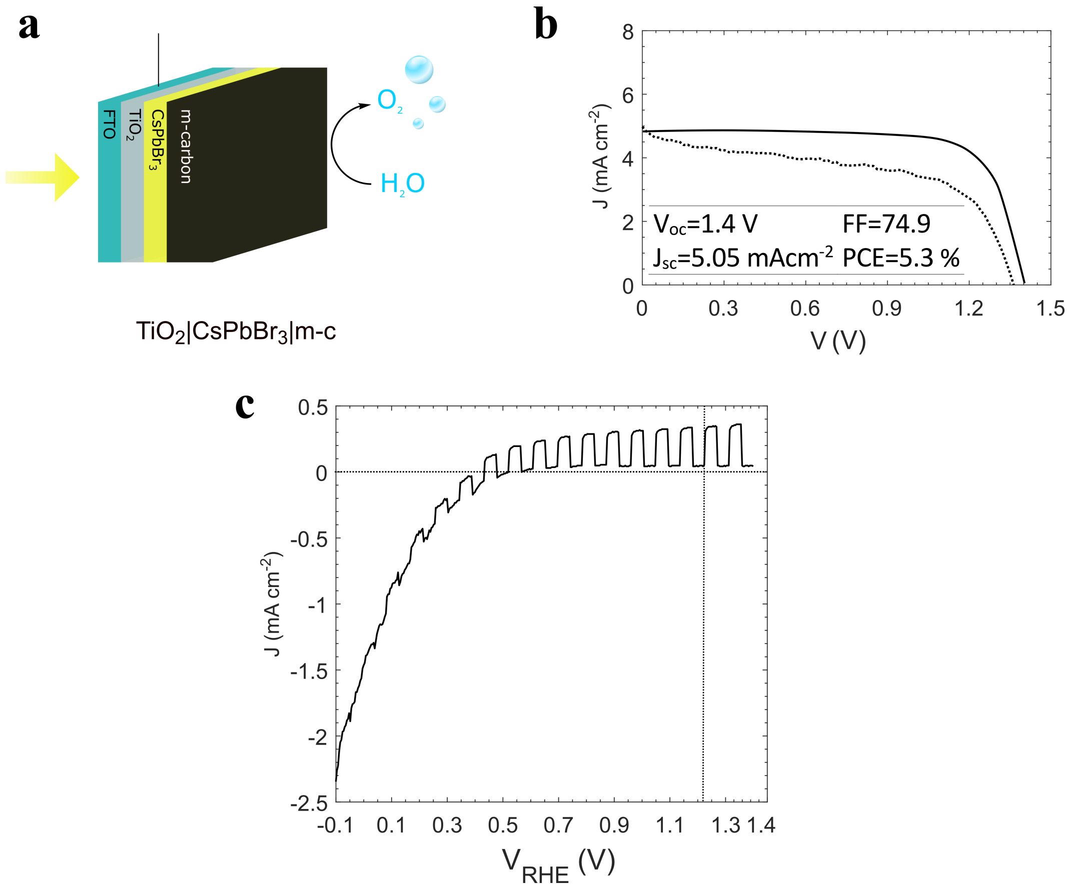

Supplementary Figure 5: TiO_2|$CsPbBr_3$|m-c as solar cell and photoanode. a Schematic illustrations of the $CsPbBr_3$ photoanode for PEC O_2 evolution: TiO_2|$CsPbBr_3$|m-c. **b** Current density-voltage curve measured as a solar cells under simulated AM 1.5 G solar light ($100\,\mathrm{mW\,cm^{-2}}$) before immersion in water. **c** LSV of TiO_2|$CsPbBr_3$|m-c photoanode measured under chopped simulated solar light in a buffer solution (k-borate, pH=9). (See Supplementary Note 1).

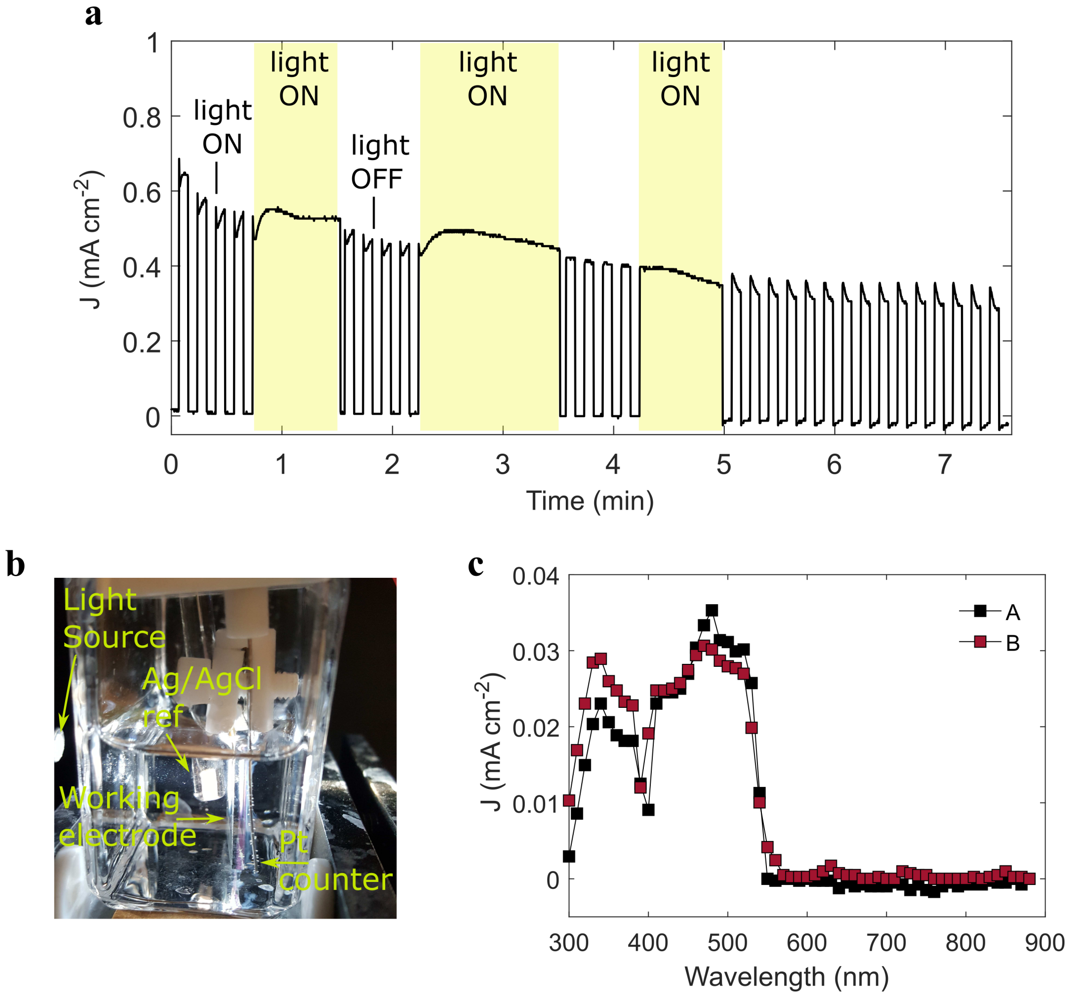

Supplementary Figure 6: $TiO_2|CsPbBr_3|$m-c photoanode stability in water. **a** PEC current density measured over time at an applied voltage of 1.23 V_{RHE} under chopped simulated solar light in buffer solution (k-borate, pH=9). **b** Picture of the three-electrode system setup used during measurement, with H_2 bubbles evolving from the Pt counter electrode. **c** PEC current density under monochromatic light measured in a buffer solution (k-borate, pH=9). Sample B was measured right after immersion while sample A was measured after it has been tested under chopped light in water for 15 min.

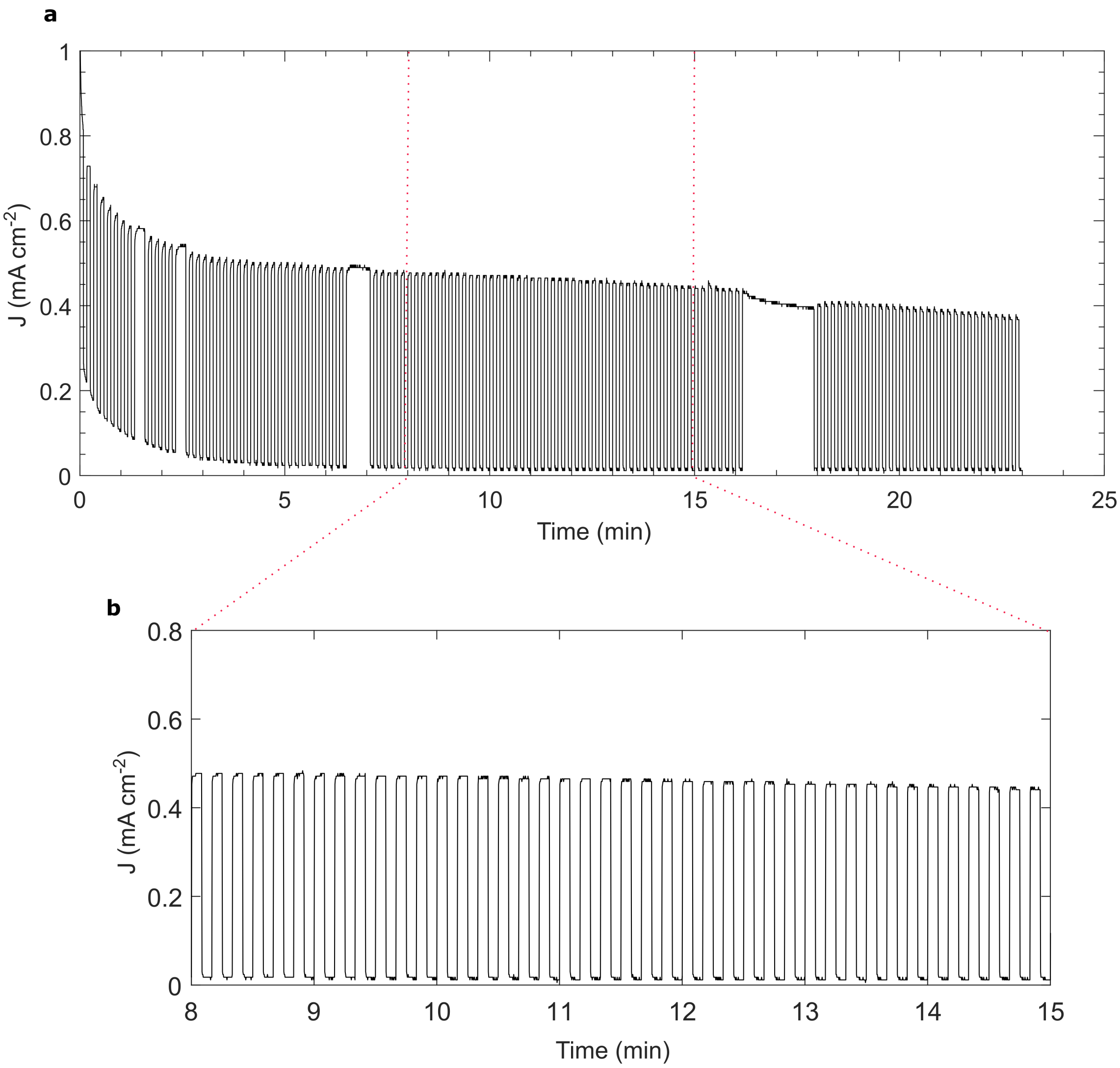

Supplementary Figure 7: TiO_2|$CsPbBr_3$|m-c photoanode stability over time. a PEC current density measured over time at an applied voltage of 1.23 V_{RHE} under chopped simulated solar light in KOH solution pH 13. **b** Magnification of the response under chopped light at minutes 8-15.

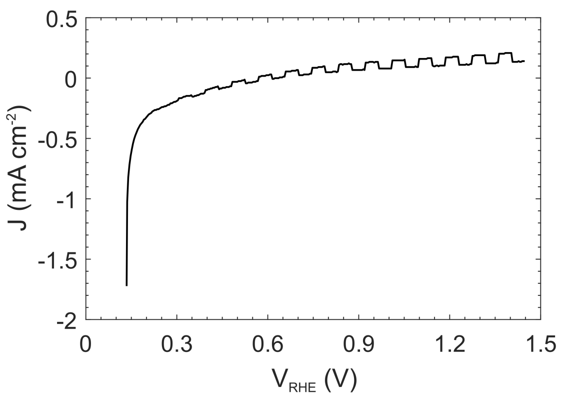

Supplementary Figure 8: TiO_2|$PbBr_2$|m-c as photoanode. LSV of TiO_2|$PbBr_2$|m-c measured under chopped simulated solar light in a buffer solution with pH=9.

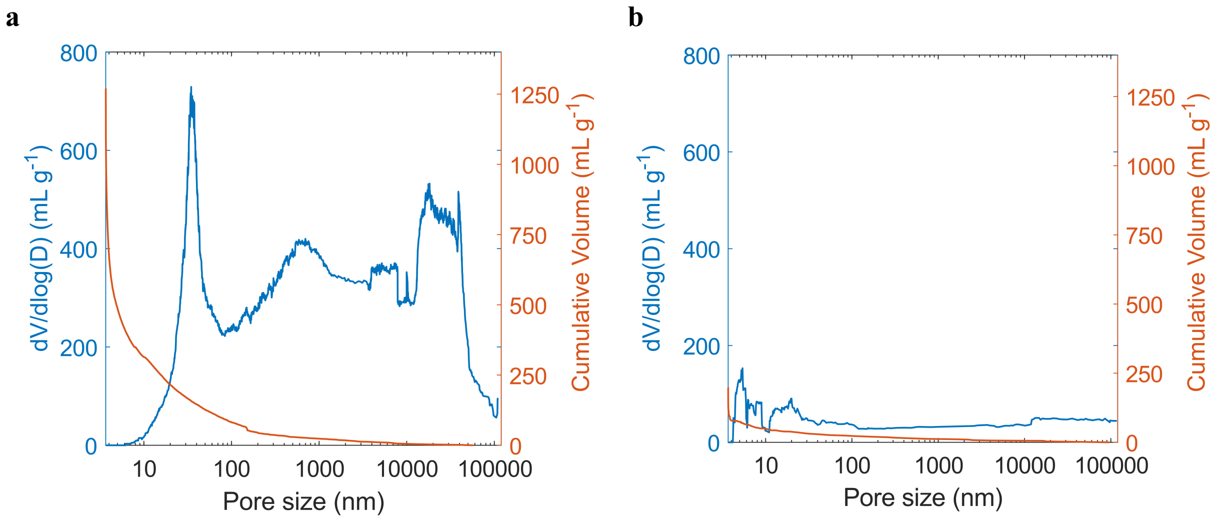

Supplementary Figure 9: Porosity of m-c and GS. Hg porosimetry of **a** m-c and **b** GS showing differences in porosity. (See Supplementary Note 2).

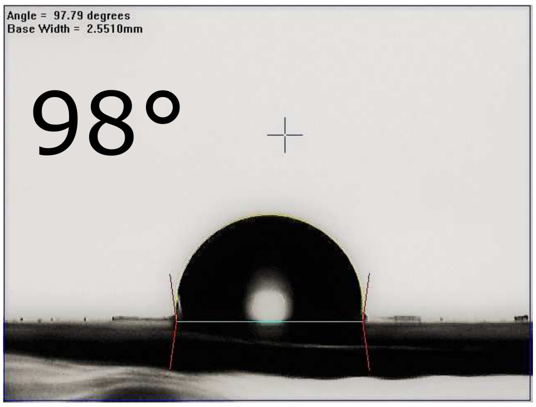

Supplementary Figure 10: Hydrophobicity of GS. Water contact angle of GS.

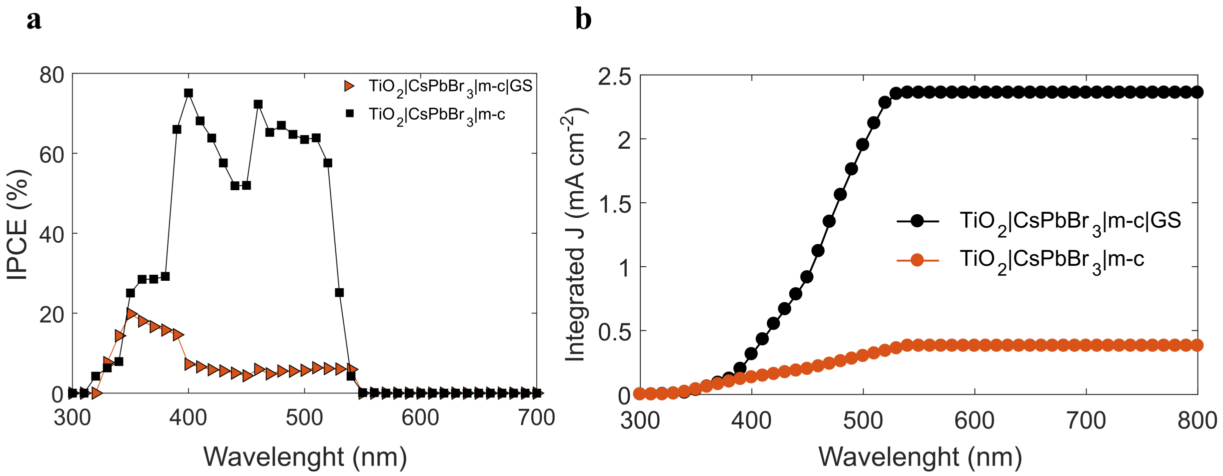

Supplementary Figure 11: Effect of GS encapsulation on PEC performance. a Wavelength dependence of the IPCE for TiO_2|$CsPbBr_3$|m-c and TiO_2|$CsPbBr_3$|m-c|GS photoanodes in aqueous buffer solution (k-borate, pH 9) under monochromatic light irradiation at $1.23\,V$ versus RHE. **b** Theoretical integrated photocurrent density obtained from the IPCE measurement.

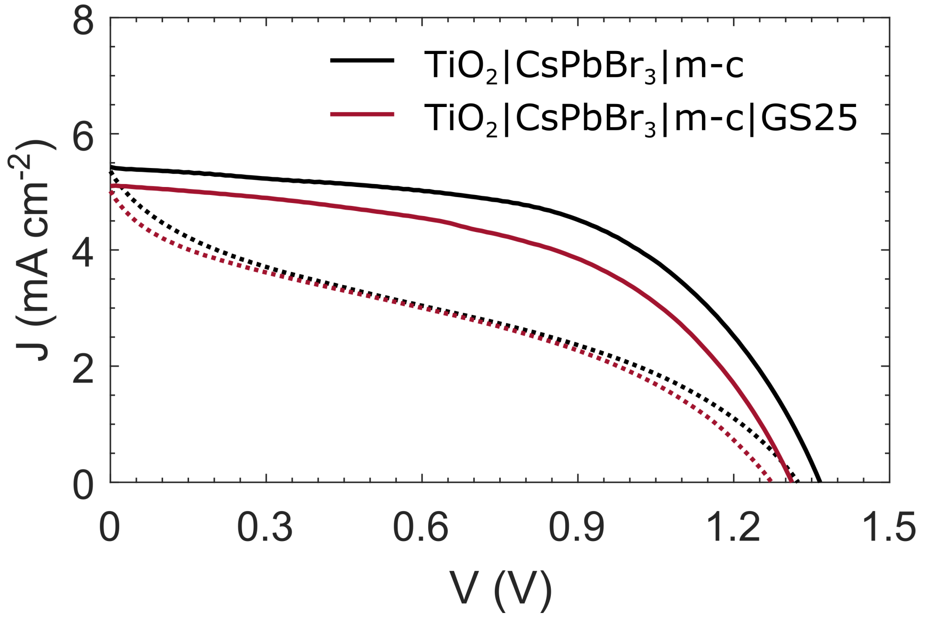

Supplementary Figure 12: Effect of GS25 on photovoltaic proerties. JV curves measured under reverse (solid line) and forward scan (dotted line) of TiO_2|$CsPbBr_3$|m-c (black line) and TiO_2|$CsPbBr_3$|m-c|GS (red line). The same device was measured before and after applying the GS on the surface. Supplementary table 1 lists the main photovoltaic parameters with the relative percentage decrease after GS was applied on the surface.

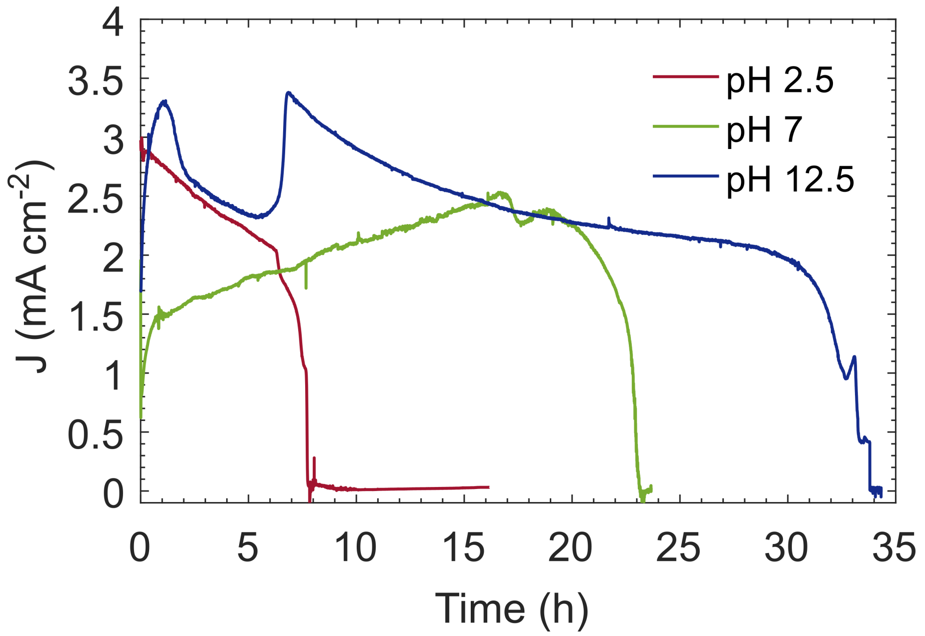

Supplementary Figure 13: Stability as a function of the pH. Chronoamperometric trace of TiO_2|$CsPbBr_3$|m-c|GS recorded at an applied potential of $1.23\,V_{RHE}$. 0.1 M KNO_3 electrolyte solution at pH adjusted to 2.5, 7 and 12.5 with H_2SO_4 and KOH, under continuous simulated solar light irradiation (AM 1.5 G, $100\,mW\,cm^{-2}$).

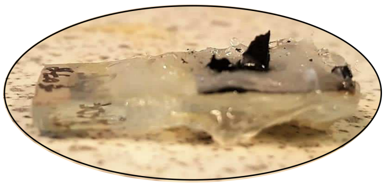

Supplementary Figure 14: Fracture of GS causing water infiltration. Photograph of the device tested at an applied potential of $1.23\,\mathrm{V}$ versus RHE. 0.1 M KNO_3 electrolyte solution pH 7, under continuous simulated solar light irradiation after $23\,\mathrm{h}$ of operation under continuous illumination.

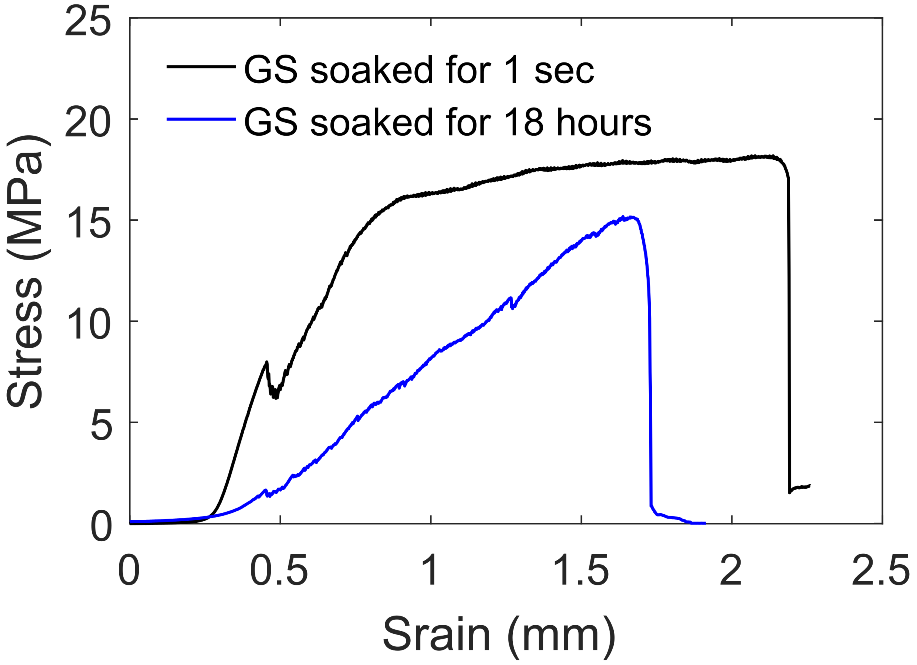

Supplementary Figure 15: Effect of soaking on the mechanical properties of GS. Tensile strength measured for wet GS; The black line indicates GS soaked in aqueous electrolyte for only $1\,\mathrm{s}$. The blue line instead indicates a GS that has been immersed in the aqueous electrolyte (0.1 M KNO_3, pH 7) for $18\,\mathrm{h}$.

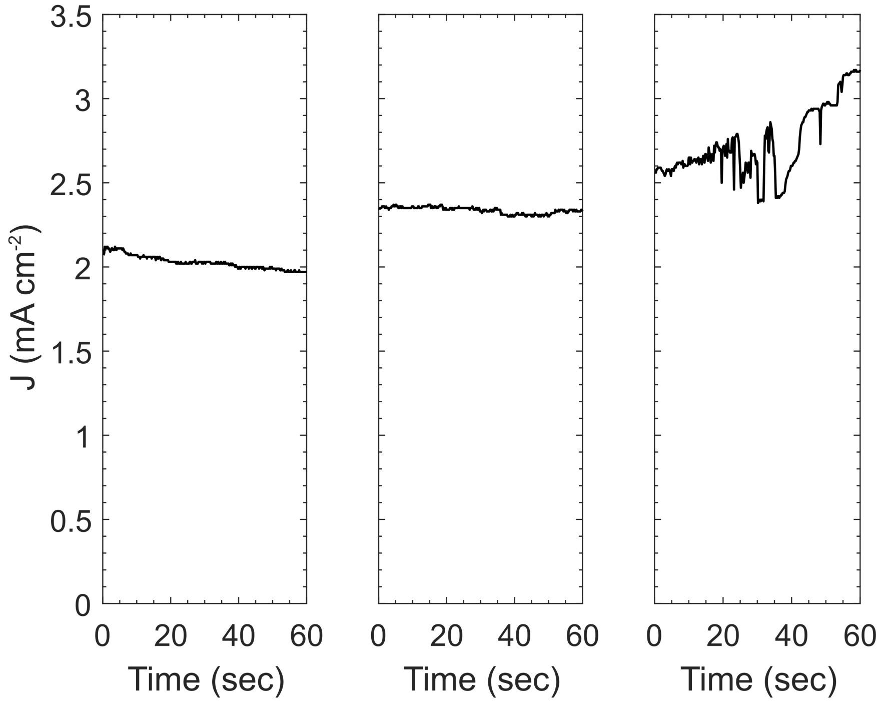

Supplementary Figure 16: Effect of soaking on the photocurrent density. Chronoamperometric trace of TiO_2|$CsPbBr_3$|m-c|GS recorded in 0.1 M KNO_3 solution (pH 7) with an applied potential of 1.23 V_{RHE}. Between each measurement the device was kept in the electrolyte solution for 1 hour in the dark at open circuit.

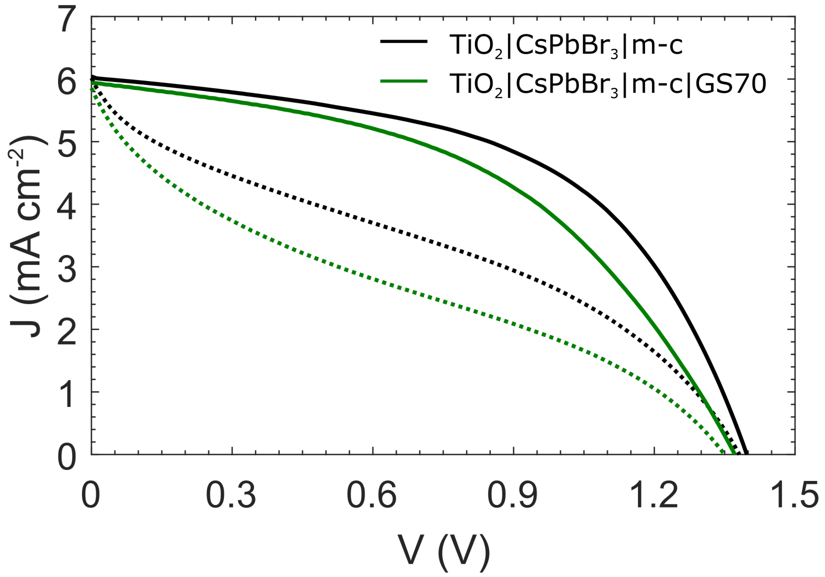

Supplementary Figure 17: Effect of GS70 on the photovoltaic properties. JV curves measured under reverse (solid line) and forward scan (dotted line) of TiO_2|$CsPbBr_3$|m-c (black line) and TiO_2|$CsPbBr_3$|m-c|GS70 (green line). The same device was measured before and after applying the GS70 on the surface. Supplementary table 2 lists the main photovoltaic parameters with the relative percentage decrease after GS70 was applied on the surface.

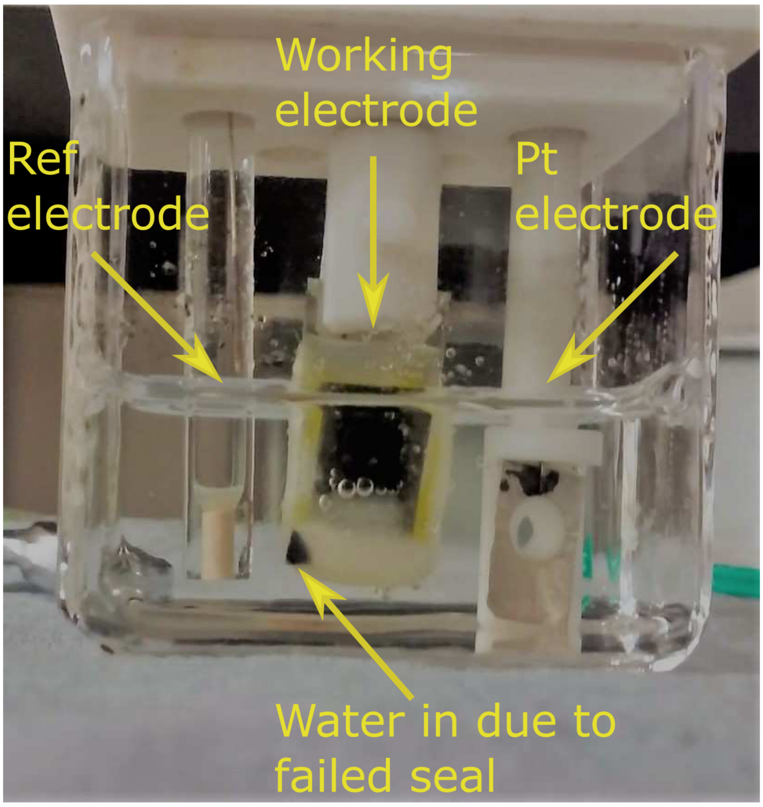

Supplementary Figure 18: PEC cell with three electrode configuration. Photograph of the three-electrode PEC cell with TiO_2|$CsPbBr_3$|m-c|GS70 photoanode used as working electrode. Water got through the device layers, dissolving the absorber material due to a leakage through the seal after the silicone-epoxy resin partially degraded in the electrolyte because of the long time immersion in water.

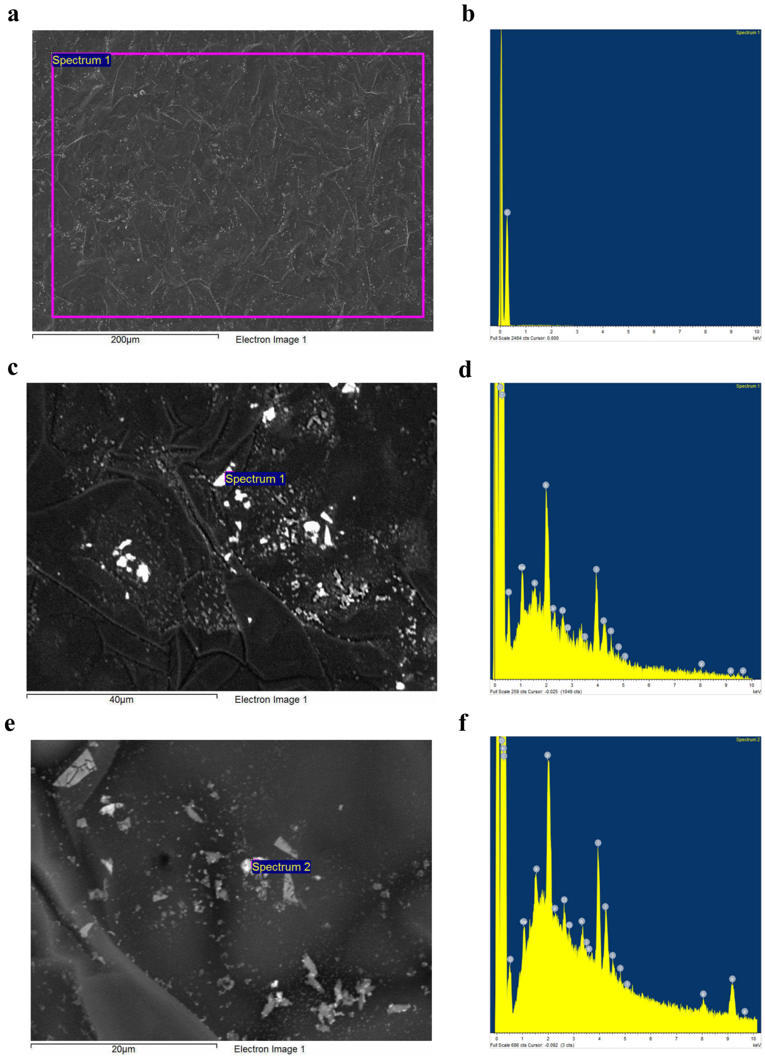

Supplementary Figure 19: Elemental composition of GS|WOC. EDX mapping images and elemental composition of GS|WOC, confirming the presence of Ir on the surface. Top-view SEM images were taken at different magnification. **a** scale bar 200 µm and **b** elemental composition of the area delimited by magenta borders in a (labelled Spectrum 1). **c** scale bar 40 µm and **d** elemental composition of the area delimited by magenta borders in c (labelled Spectrum 1). **e** scale bar 20 µm and **f** elemental composition of the area delimited by magenta borders in e (labelled Spectrum 2).

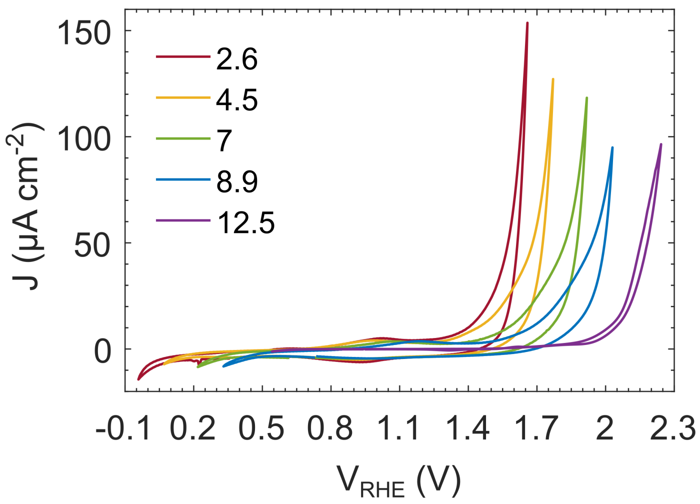

Supplementary Figure 20: Effect of pH on the water oxidation of GS|WOC. CV scans of GS|WOC electrodes performed in 0.1 M KNO_3 solution with pH adjusted with H_2SO_4 and KOH; scan rate of $50\,\mathrm{mV\,s^{-1}}$

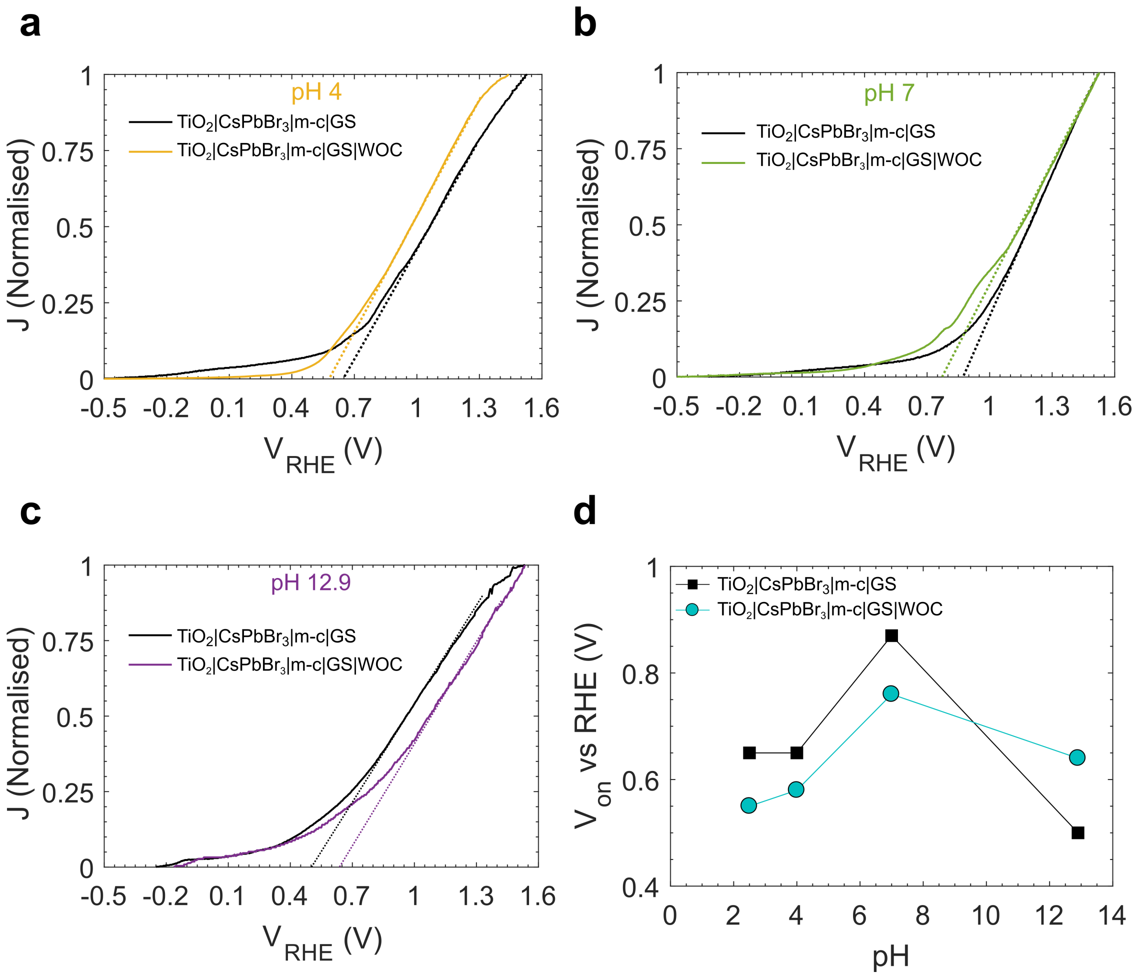

Supplementary Figure 21: Effect of pH on the onset potential. LSV of TiO_2|$CsPbBr_3$|m-c|GS photoanodes with and without functionalised GS measured in 0.1 M KNO_3 with pH adjusted with H_2SO_4 and KOH at **a** 4, **b** 7 and **c** 12.9. The photocurrent density has been normalised with respect to the maximum value obtained. **d** Onset potentials of TiO_2|$CsPbBr_3$|m-c|GS (GS) and TiO_2|$CsPbBr_3$|m-c|GS|WOC (GS|WOC) photoanodes as a function of the pH. (See Supplementary Note 3).

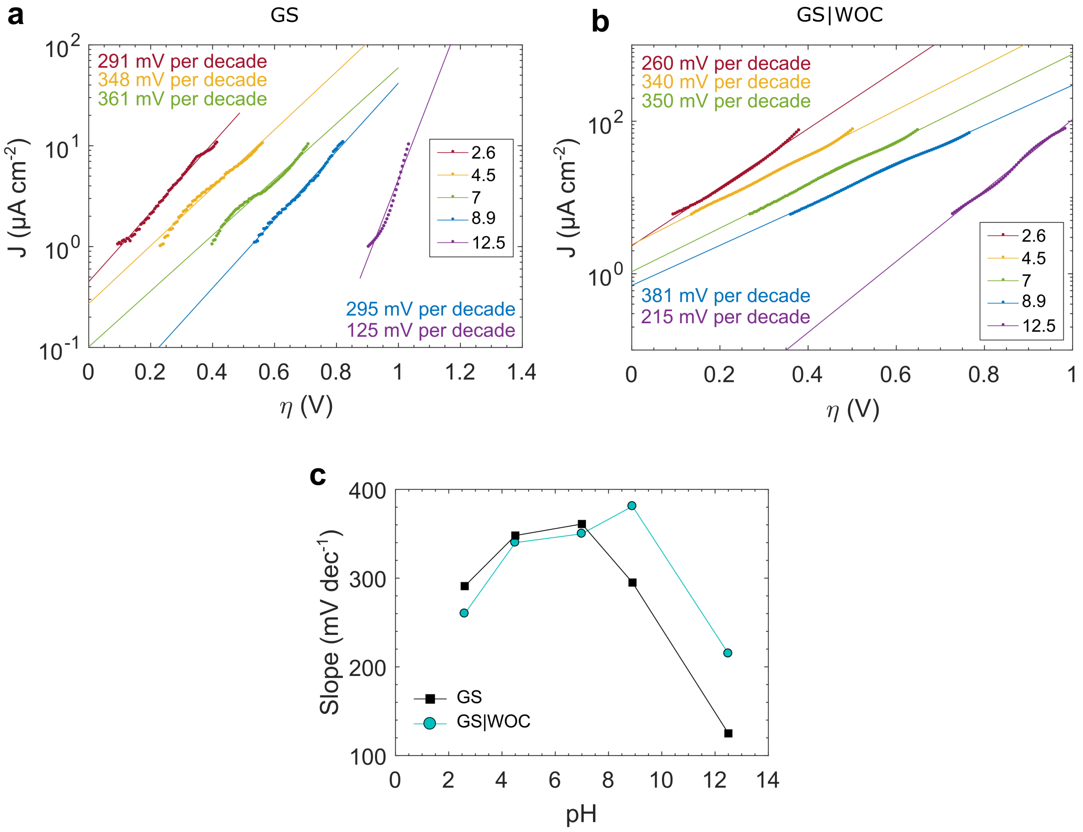

Supplementary Figure 22: Tafel analysis of GS|WOC. Tafel plots of **a** GS and **b** GS|WOC derived from the cyclic voltammograms displayed in Fig 20. **c** Tafel slopes of GS and GS|WOC electrodes as a function of pH of the solution

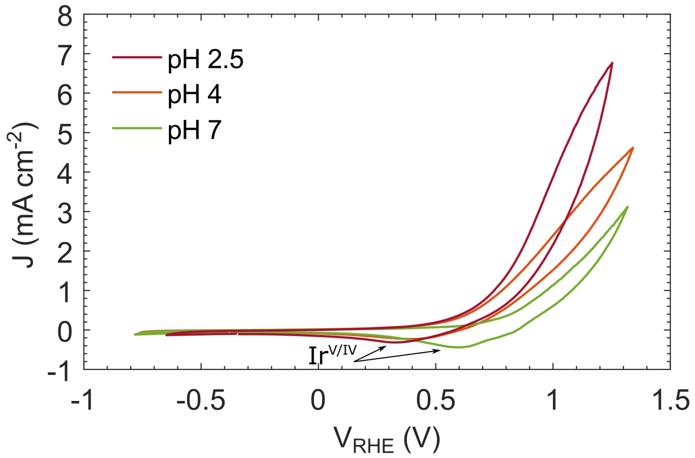

Supplementary Figure 23: Effect of pH on the TiO_2|$CsPbBr_3$|m-c|GS|WOC PEC performance. CV scans of TiO_2|$CsPbBr_3$|m-c|GS|WOC electrodes performed in 0.1 M KNO_3 solution at pH 2.5, 4 and 7 with a scan rate of $50\,\mathrm{mV\,s^{-1}}$. The reduction wave for the $Ir^{V/IV}$ couple can be seen on the reverse scan, while it is not detected in the forward scan. The same behaviour was previously observed on hematite photoanodes functionalised with the same Ir-WOC. [1]

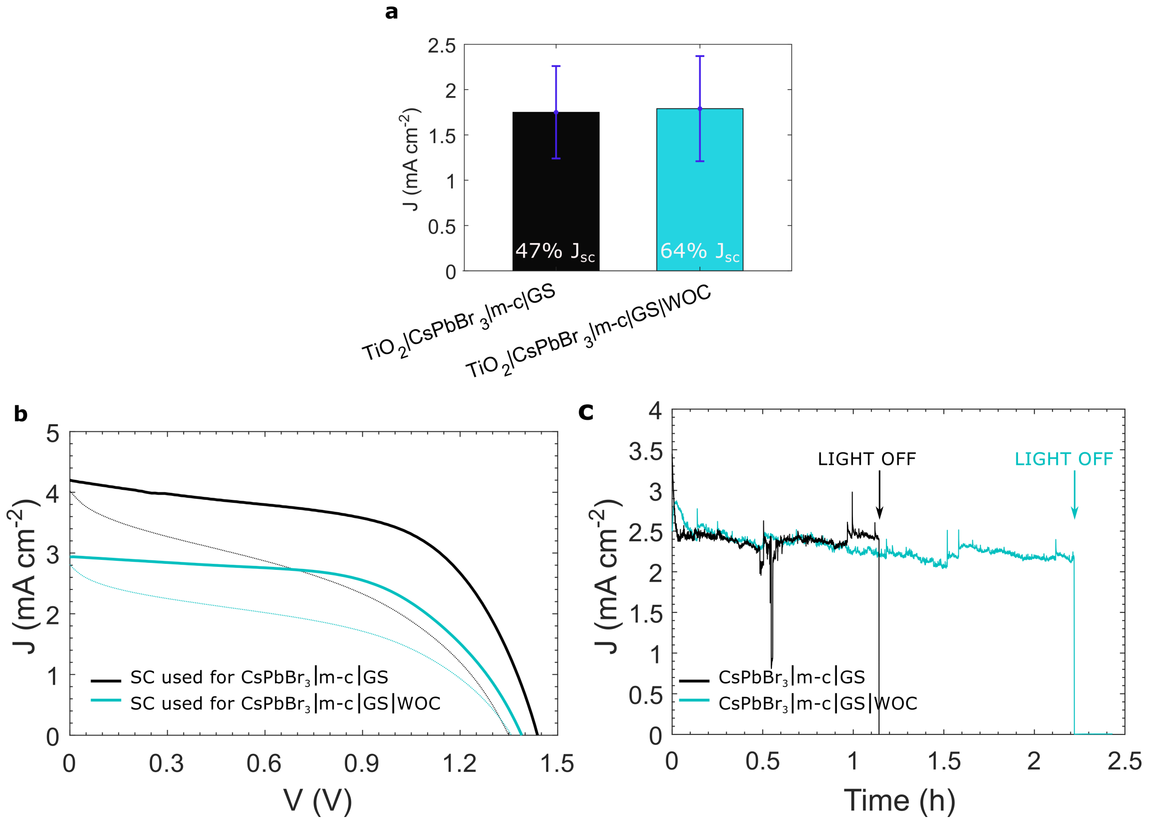

Supplementary Figure 24: PV and PEC performance of TiO_2|$CsPbBr_3$|m-c|GS devices. **a** PEC current density measured at 1.23 V versus RHE averaged over 11 and 7 samples respectively (error bars indicate the standard deviation). **b** JV curves of the as-prepared TiO_2|$CsPbBr_3$|m-c solar cells used to prepare the photoanodes for PEC testing. The solid line is measured under reverse scan. The dotted line is measured under forward scan. **c** Chronoamperometric traces of the resulting photoanodes TiO_2|$CsPbBr_3$|m-c|GS and TiO_2|$CsPbBr_3$|m-c|GS|WOC recorded in 0.1 M KNO_3 with pH adjusted to 3.5 with H_2SO_4 under constant illumination. (See Supplementary Note 4).

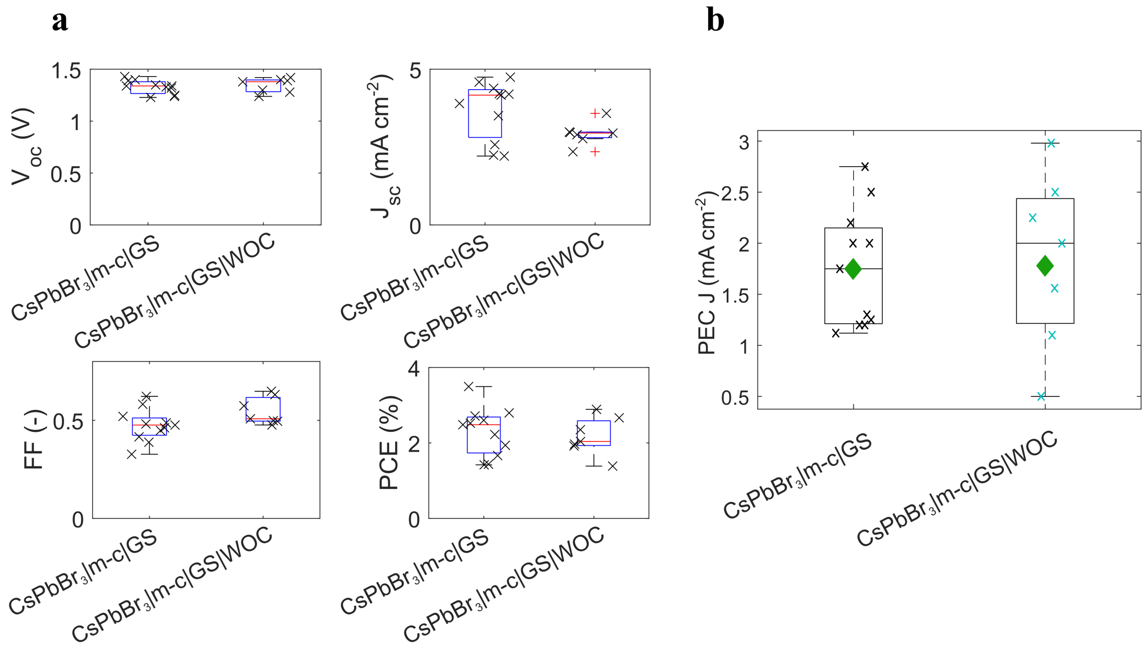

Supplementary Figure 25: Statistical analysis of PV and PEC performance of TiO_2|$CsPbBr_3$|m-c|GS. a Box plots of the main photovoltaic parameters of the as-prepared TiO_2|$CsPbBr_3$|m-c solar cells used to prepare the photoanodes for PEC testing. **b** Box plots of PEC J at 1.23 V versus RHE of TiO_2|$CsPbBr_3$|m-c|GS and TiO_2|$CsPbBr_3$|m-c|GS|WOC. The green markers indicate the average value. (See Supplementary Note 4).

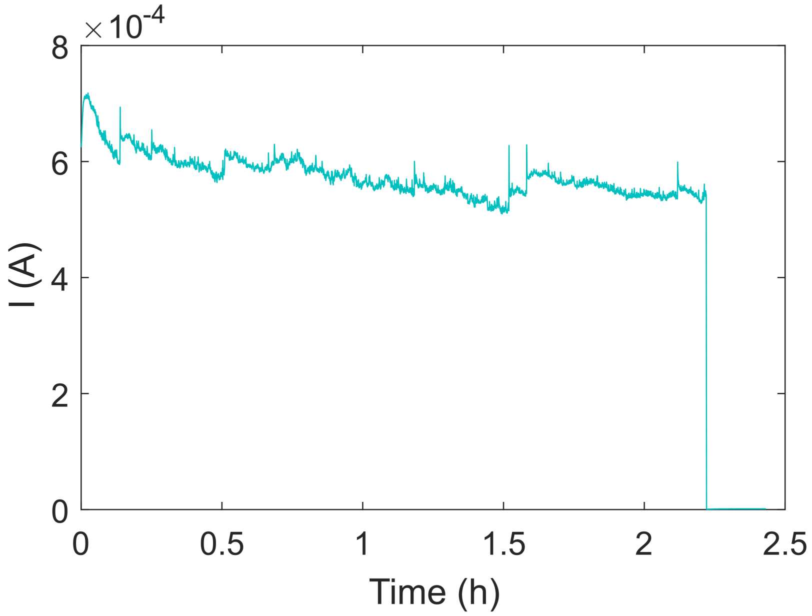

Supplementary Figure 26: Stability of TiO_2|$CsPbBr_3$|m-c|GS|WOC in three-electrode configuration. Chronoamperometric trace of TiO_2|$CsPbBr_3$|m-c|GS|WOC recorded in 0.1 M KNO_3 with pH adjusted to 3.5 with H_2SO_4 under continuous simulated solar light illumination during O_2 evolution detection. (See Supplementary Note 5).

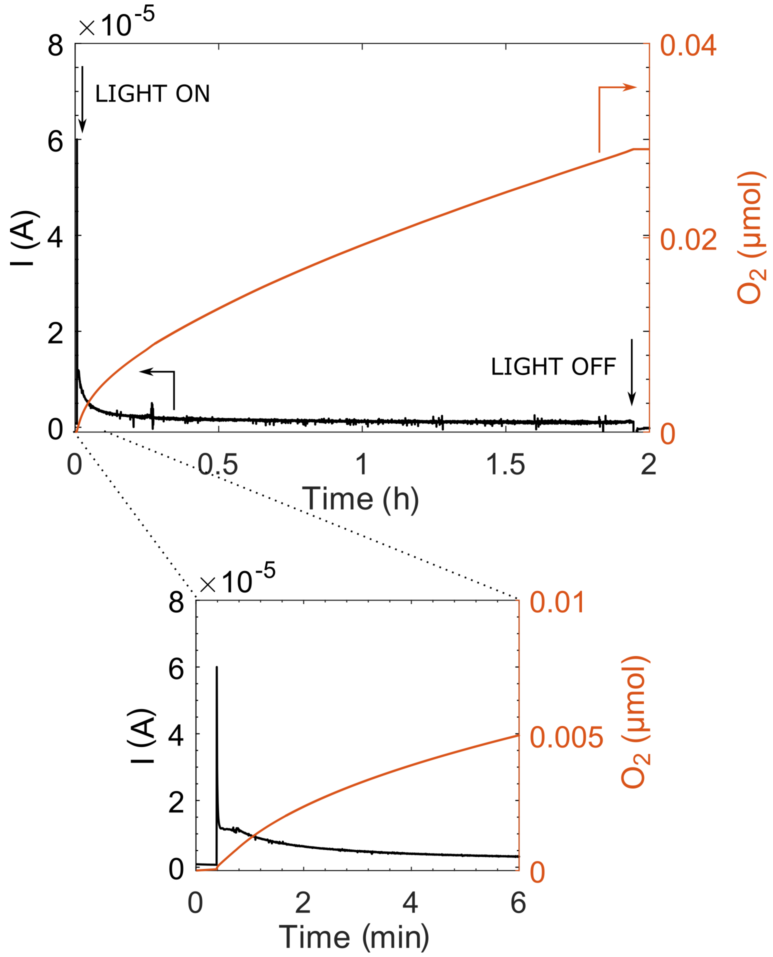

Supplementary Figure 27: Stability of TiO_2|$CsPbBr_3$|m-c|GS|WOC in two-electrode configuration. Chronoamperometric trace of TiO_2|$CsPbBr_3$|m-c|GS|WOC recorded in a two-electrode cell in 0.1 M KNO_3 (pH adjusted to 2.5 with H_2SO_4) without applying any external bias between the working and Pt counter electrode and predicted O_2 production, assuming that O_2 formation liberates 4 electrons.

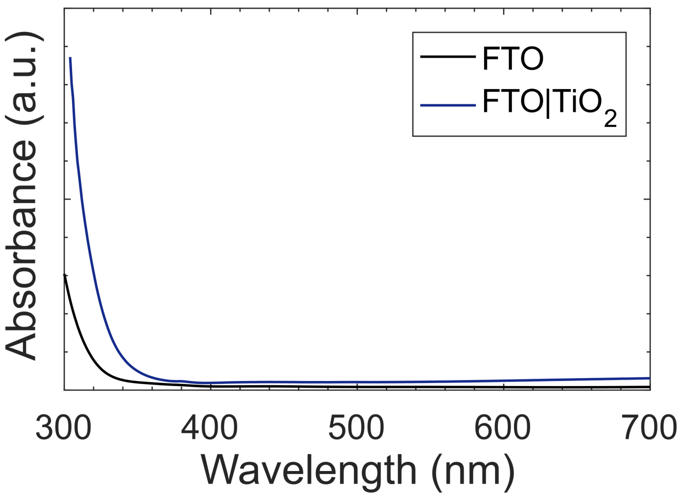

Supplementary Figure 28: Absorbance of FTO c-glass substrates. UV-Vis absorbance of glass coated with FTO and FTO|TiO_2

Supplementary Tables

Supplementary Table 1: Effect of GS25 on the main photovoltaic parameters. Photovoltaic parameters of the $TiO_2/CsPbBr_3$/m-c solar cell shown in Supplementary Figure 12 before and after applying the GS25 on top of m-c layer.

	**$TiO_2/CsPbBr_3$/m-c**	**$TiO_2/CsPbBr_3$/m-c/GS**	% decrease
J_{sc} ($mA\,cm^{-2}$)	5.43	5.12	5.7
V_{oc} (V)	1.36	1.31	3.7
FF	0.56	0.53	5.3
PCE (%)	4.13	3.50	15.2

Supplementary Table 2: Effect of GS70 on the main photovoltaic parameters. Photovoltaic parameters of the TiO_2/$CsPbBr_3$/m-c solar cell shown in Supplementary Figure 17 before and after applying the GS70 on top of m-c layer.

	**TiO_2/$CsPbBr_3$/m-c**	**TiO_2/$CsPbBr_3$/m-c/GS70**	% decrease
J_{sc} ($\mathrm{mA\,cm^{-2}}$)	6.04	5.94	1.7
V_{oc} (V)	1.39	1.37	1.4
FF	0.53	0.47	11.3
PCE (%)	4.47	3.85	13.9

Supplementary Notes

Supplementary Note 1 Supplementary Fig. 5: Since electrolytes were exposed to air, oxygen reduction taking place at the FTO electrodes explains the dark negative current at cathodic potentials. [2] Indeed, when all the photoanode but the active area was covered with epoxy, avoiding physical contact between the FTO substrate and the electrolyte solution, the dark current at negative potentials was substantially reduced. Similar dark negative currents have been reported in literature [3–5] and they are not particularly relevant to the performance of the photoanode that was studied at higher applied potentials of $1.23\,\mathrm{V}_{RHE}$, where the dark current is in fact zero.

Supplementary Note 2 Supplementary Fig. 9: The porosity of m-c and GS have been measured via mercury porosimetry analysis, which studies the progressive intrusion of mercury into a porous structure under stringently controlled pressures (going from low to high penetration pressure). This technique allows to determine the pore size of the material. The steep part of the curve in Fig. 9a represents the mercury penetration in well-defined pores. The mesoporous carbon layer m-c is highly porous. In contrast, GS is highly compact and dense and has a very poor porosity.

Supplementary Note 3 Supplementary Fig. 21d: The onset potential V_{on} of TiO_2|$CsPbBr_3$|m-c|GS and TiO_2|$CsPbBr_3$|m-c|GS|WOC has been measured in electrolytes with pH ranging between 2.5 and 12. Herein, V_{on} is defined conservatively as the potential that corresponds to the intercept between the dark current and the tangent to the maximum slope of the current rise under illumination. [6]

Supplementary Note 4 Supplementary Fig. 24 and Supplementary Fig. 25: Average PEC current density J measured on 11 TiO_2|$CsPbBr_3$|m-c|GS and 7 TiO_2|$CsPbBr_3$|m-c|GS|WOC photoanodes at $1.23\,V_{RHE}$. Measurements were taken from distinct samples. The PEC J at $1.23\,V_{RHE}$ of TiO_2|$CsPbBr_3$|m-c|GS was on average $47\,\%$ of J_{sc} of the as-prepared solar cells, while PEC J of TiO_2|$CsPbBr_3$|m-c|GS|WOC was $64\,\%$ of J_{sc} of the as-prepared solar cells. The average water oxidation photocurrents are equal but the average photovoltaic currents (Jsc) of TiO_2|$CsPbBr_3$|m-c|GS|WOC devices were lower than the ones measured for TiO_2|$CsPbBr_3$|m-c|GS.
Box plots in Supplementary Fig 25: The boxes plot the first and third quartiles, and the band inside the box is the second quartile (the median). The ends of the whiskers represent that the lowest datum is still within 1.5 of the interquartile range (IQR) of the lower quartile, and the highest datum is still within 1.5 IQR of the upper quartile. Red crosses indicate outlier data that are out of the 1.5 IQR.

Supplementary Note 5 The O_2 evolution on the $TiO_2|CsPbBr_3|$m-c$|$GS$|$WOC under constant illumination at 1.23 V versus RHE was measured. Supplementary Fig. 26 shows the chronoamperometric trace recorded during O_2 evolution detection, which was used to calculate the predicted O_2 production.

Supplementary Discussion

The predicted O_2 value was compared with the measured oxygen level to calculated the Faradaic efficiency. A Faradaic efficiency of $80.9\,\%$ was achieved after $2\,\mathrm{h}$ of testing. Part of the generated oxygen will dissolve in the solution (which is purged for $15\,\mathrm{minute}$ with N_2 before the start of the experiment) and therefore would not be detected by an oxygen probe in the headspace of the cell. The oxygen dissolved in the liquid was estimated according to Henry's law.

The observed delay between the generation of O_2 at the photoanode surface and the actual detection in the headspace of the cell can be assigned to the slow diffusion of oxygen into the membrane of the electrode sensor. [7] In fact, before reaching the probe and be detected, oxygen has to diffuse through any porosity in the graphite layers, saturate the electrolyte, diffuse in the headspace, and finally diffuse through the sensor membrane.

Supplementary References

1. Moir, J. W., Sackville, E. V., Hintermair, U. & Ozin, G. A. Kinetics versus Charge Separation: Improving the Activity of Stoichiometric and Non-Stoichiometric Hematite Photoanodes Using a Molecular Iridium Water Oxidation Catalyst. *J. Phys. Chem. C* **120**, 12999–13012 (2016).

2. Díez-García, M. I. *et al.* $YFeO_3$ Photocathodes for Hydrogen Evolution. *Electrochim. Acta* **246**, 365–371 (2017).

3. Gurudayal *et al.* Core-Shell Hematite Nanorods: A Simple Method To Improve the Charge Transfer in the Photoanode for Photoelectrochemical Water Splitting. *ACS Appl. Mater. Interfaces* **7**, 6852–6859 (2015).

4. Bu, Y. *et al.* Optimization of the Photo-Electrochemical Performance of Mo-Doped $BiVO_4$ Photoanode by Controlling the Metal-Oxygen Bond State on (020) Facet. *Adv. Mater. Interfaces* **4**, 1601235 (2017).

5. Kumar, P. *et al.* Quantum dot activated indium gallium nitride on silicon as photoanode for solar hydrogen generation. *Commun. Chem.* **2**, 4 (2019).

6. Cao, D. *et al.* Cathodic shift of onset potential for water oxidation on a Ti^{4+} doped Fe_2O_3 photoanode by suppressing the back reaction. *Energy and Environmental Science* **7**, 752–759 (2014).

7. Shi, Y. *et al.* CuO-Functionalized Silicon Photoanodes for Photoelectrochemical Water Splitting Devices. *ACS Appl. Mater. Interfaces* **8**, 695–702 (2016).
